# Superior Effects of High-Intensity Interval Training vs. Moderate Continuous Training on Arterial Stiffness in Episodic Migraine: A Randomized Controlled Trial

**DOI:** 10.3389/fphys.2017.01086

**Published:** 2017-12-19

**Authors:** Henner Hanssen, Alice Minghetti, Stefano Magon, Anja Rossmeissl, Athina Papadopoulou, Christopher Klenk, Arno Schmidt-Trucksäss, Oliver Faude, Lukas Zahner, Till Sprenger, Lars Donath

**Affiliations:** ^1^Department of Sport, Exercise and Health, University of Basel, Basel, Switzerland; ^2^Department of Neurology, University Hospital Basel and University of Basel, Basel, Switzerland; ^3^Medical Image Analysis Center, University Hospital Basel, Basel, Switzerland; ^4^Department of Neurology, DKD HELIOS Klinik Wiesbaden, Wiesbaden, Germany; ^5^Department of Intervention Research in Exercise Training, Institute of Exercise Training and Computer Science in Sport, German Sport University Cologne, Köln, Germany

**Keywords:** migraine, exercise, high intensity interval training, arterial stiffness, cardiovascular risk, randomized controlled trial

## Abstract

**Background:** Migraine is associated with increased cardiovascular risk and vascular dysfunction. Since aerobic exercise can reduce cardiovascular risk, the present randomized controlled trail aimed at investigating the effects of high-intensity interval training (HIT) vs. moderate continuous exercise training (MCT) on arterial stiffness in migraine patients.

**Methods:** Forty-eight episodic migraineurs were initially enrolled in the study. 37 patients [female: 30; age: 37 (*SD*: 10); BMI: 23.1 (5.2); Migraine days per month: 3.7 (2.5)] completed the intervention. Central blood pressure, pulse wave reflection, and aortic pulse wave velocity (PWV) were obtained by an oscillometric monitor. Incremental treadmill exercise testing yielded maximal and submaximal fitness parameters. Participants were randomly assigned to either HIT, MCT, or a control group (CON). The intervention groups trained twice a week over a 12-week intervention period.

**Results:** After adjustment for between-group baseline differences, a moderate meaningful overall reduction of the augmentation index at 75 min^−1^ heart rate (AIx@75) was observed [partial eta squared ($\eta_{p}^{2}$) = 0.16; *p* = 0.06]. With 91% likely beneficial effects, HIT was more effective in reducing AIx@75 than MCT [HIT: pre 22.0 (9.7), post 14.9 (13.0), standardized mean difference (SMD) = 0.62; MCT: pre 16.6 (8.5), post 21.3 (10.4), SMD −0.49]. HIT induced a relevant reduction in central systolic blood pressure [cSBP: pre 118 (23) mmHg, post 110 (16) mmHg, SMD = 0.42] with a 59% possibly beneficial effect compared to CON, while MCT showed larger effects in lowering central diastolic blood pressure [pre 78 (7) mmHg, post 74 (7) mmHg, SMD = 0.61], presenting 60% possibly beneficial effects compared to CON. Central aortic PWV showed no changes in any of the three groups. Migraine days were reduced more successfully by HIT than MCT (HIT: SMD = 1.05; MCT: SMD = 0.43).

**Conclusion:** HIT but not MCT reduces AIx@75 as a measure of pulse wave reflection and indirect marker of systemic arterial stiffness. Both exercise modalities beneficially affect central blood pressure. HIT proved to be an effective complementary treatment option to reduce vascular dysfunction and blood pressure in migraineurs.

## Introduction

Migraine is considered a debilitating neurological disease which recurs in the form of severe headache attacks accompanied with nausea, vomiting, phonophobia, and photophobia (Jiménez Caballero and Muñoz Escudero, 2013). Despite high prevalence rates (Stovner and Andree, 2010), the complex pathogenetic mechanisms involved in migraine remain to be elucidated (Jiménez Caballero and Muñoz Escudero, 2013). Various studies have been able to link migraine to adverse vascular risk profiles (Sacco et al., 2012, 2015). Migraine is associated with an increased risk of cardiovascular disease (CVD) such as myocardial infarction and stroke (Kurth et al., 2008; Schürks et al., 2009, 2011; Spector et al., 2010). A recent study revealed that there are about 2.6 million individuals in the U.S. suffering from episodic migraine with one or more cardiovascular events or conditions (Buse et al., 2017). Migraineurs have independently been associated with increased aortic stiffness and enhanced peripheral wave reflection (Schillaci et al., 2010), endothelial dysfunction (Jiménez Caballero and Muñoz Escudero, 2013) as well as increased hypercoagulability and inflammation (Kurth et al., 2008). This evidence suggests that functional properties of large arteries are altered in migraine patients (Liman et al., 2012), leading to an overall increase in cardiovascular risk (Liew et al., 2006; Rose et al., 2007; Kurth et al., 2008, 2016; Buse et al., 2017).

Arterial stiffness is an established independent predictor of cardiovascular events and stroke in healthy patients (Mitchell et al., 2010). The augmentation index (AIx) serves as an established systemic haemodynamic marker of pulse wave reflection and is closely related to the development of atherosclerosis and incidence cardiovascular events (Vlachopoulos et al., 2010a). An increased AIx displays a greater contribution of the reflected wave to the central blood pressure, indicating increased arterial stiffness and cardiac afterload as well as an unfavorably altered central blood pressure. As the AIx is dependent on heart rate (HR) (Wilkinson et al., 2000), the corrected augmentation index calculated for a set of heart rate of 75 beats per minute (AIx@75) is usually applied for a more valid comparison in resting state. Pulse wave velocity (PWV) is a direct measure of large artery stiffness and an independent predictor of CV morbidity and mortality (Hansen, 2010; Vlachopoulos et al., 2010b; Ben-Shlomo et al., 2014).

Higher levels of physical activity and cardiorespiratory fitness reduce all-cause mortality and CVD (Rognmo et al., 2004). Physical activity inversely correlates with an age-dependent increase of arterial stiffness (Kozakova et al., 2007) and cardiorespiratory fitness and sports-related activity are inversely correlated with arterial stiffness in younger adults (Boreham et al., 2004). More recently, higher levels of moderate-to-vigorous physical activity were independently associated with slower age-related progression of central arterial stiffness (Ahmadi-Abhari et al., 2017). Furthermore, endurance exercise training has been shown to improve arterial stiffness in patients with coronary artery disease (Edwards et al., 2004). Regular high-intensity interval training (HIT) has been shown to induce superior improvement in aerobic fitness, endothelial function, as well as other cardio-metabolic risk factors in comparison to moderate continuous exercise training (MCT) (Wisløff et al., 2007; Tjønna et al., 2008). Higher exercise intensities applied in intermittent bouts of highly intense aerobic exercise seem to affect pulse wave reflection more beneficially than moderate continuous exercise (Hanssen et al., 2015). To date, few studies have examined the effects of different exercise modalities in patients suffering from migraine (Hanssen et al., 2017). This is the first study comparing the effects of different longer term exercise training modalities on arterial stiffness and attack frequency in migraineurs. We hypothesized that regular HIT is a more beneficial means compared to MCT for the reduction of central and peripheral arterial stiffness indices, blood pressure, and migraine attack frequency.

## Methods

### Study design and participants

**Figure 1:**
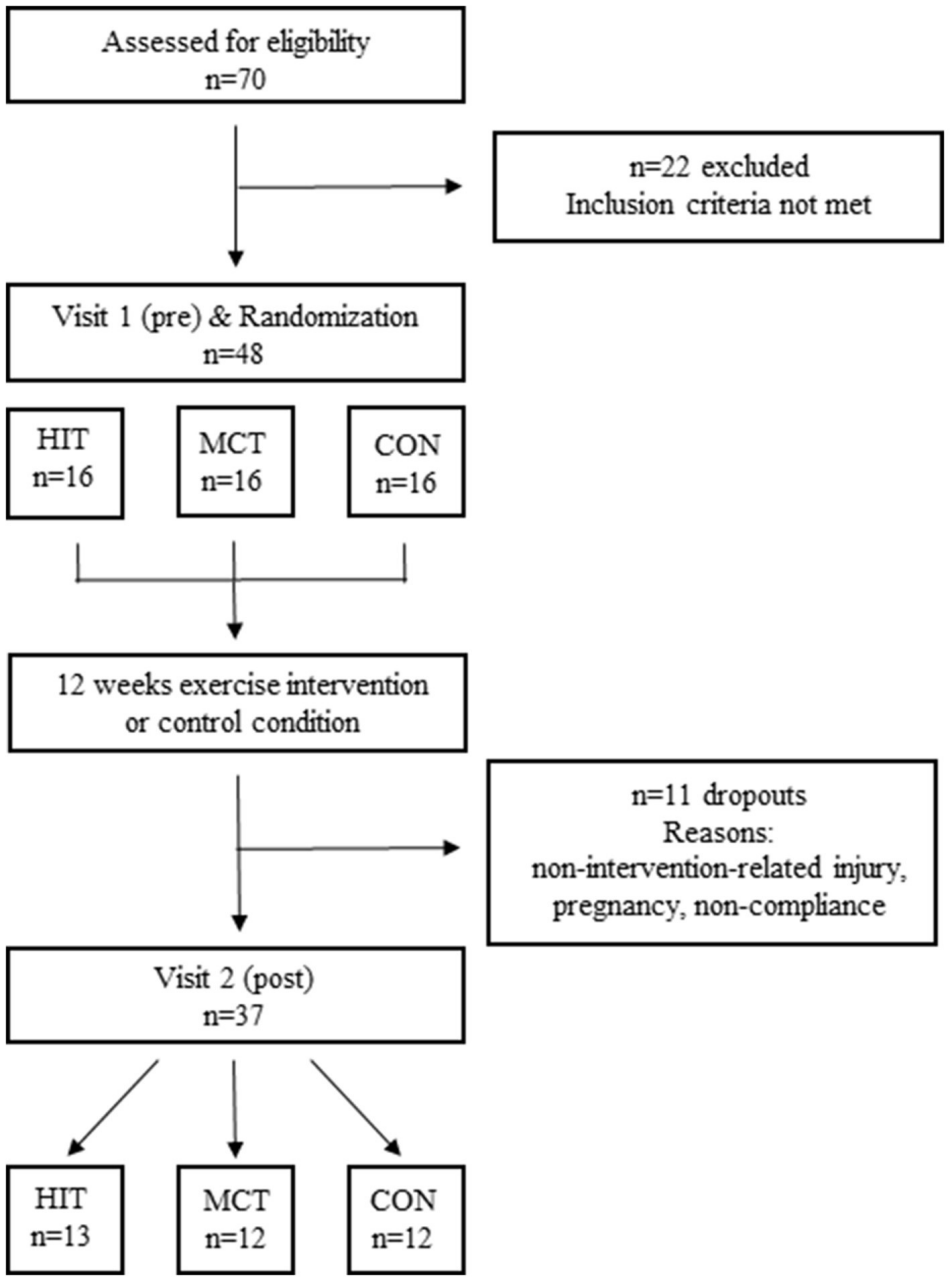
Flow-chart of the randomized controlled trial.

The present study was designed as a three-armed randomized controlled trial with a primary endpoint set on the effect of exercise training on pulse wave reflection in patients with migraine. In order to determine a clinical baseline for disease severity, patients underwent a 4-week run-in period prior to the start of the intervention period. After the run-in period and pre-testing measurements, 48 patients were randomly assigned [minimization method (Pocock and Stone, 2016), strata: age, gender, BMI, PA, migraine assessment according to MIDAS score, physical fitness determined by VO_2_max] to one of three groups: high intensity aerobic interval training group (HIT), moderate continuous aerobic training group (MCT), or control group (CON). Baseline parameters are depicted in Table 1. The intervention groups MCT and HIT trained twice a week over the 12-week intervention period. CON were requested to maintain their habitual daily physical activity profile and received additional standard physical activity recommendations. During the 12 weeks, a total of 11 (23%) patients dropped out due to injury, lack of motivation or personal reasons (Figure 1). Before and after 12 weeks of training, pulse wave reflection, PWV, and cardiopulmonary exercise testing were assessed in pre- and post-testing procedures. During the entire training study, patients were asked to keep a migraine and physical activity diary documenting the frequency and side effects of the attacks as well as their physical activity profiles. The study was registered in the German clinical trial register (DRKS-ID: DRKS00008015) and has been approved by the regional ethics committee (Ethical approval number: 194/13). All subjects signed an informed written consent after receiving all relevant study information.

**Table 1:** Baseline data of the participants for both intervention groups (HIT and MCT) and the control group (CON).

	**HIT** (n = 13)	**MCT** (n = 12)	**CON** (n = 12)
Gender [m/f]	3/10	2/10	2/10
Age [years]	36.2 (10.7)	36.5 (8.7)	37.3 (11.9)
BMI [kg·m^−2^]	22.4 (3.0)	23.6 (9.7)	23.4 (2.8)
Systolic BP [mmHg]	118.1 (23.4)	109.8 (9.1)	113.7 (10.8)
Diastolic BP [mmHg]	78.7 (5.9)	77.8 (6.8)	78.3 (7.6)
FFKA_MET [MET/week]	36.5 (50.8)	38.9 (37.0)	35.1 (20.6)
MIDAS [score]	21.4 (13.4)	24.0 (20.9)	16.3 (8.9)

*Data are provided as means with standard deviations (SD). Gender is indicated as f, female; m, male; BMI, body mass index; BP, blood pressure; HR, resting heart rate; FFKA_MET, Freiburger Physical Activity Questionnaire expressed in metabolic equivalents per week; MIDAS, Migraine Disability Assessment Questionnaire expressed in MIDAS score*.

All examinations were performed at the University of Basel, Switzerland. Patients were recruited from the outpatient division of the Department of Neurology at the University Hospital Basel and via advertisements. Following the initial clinical screening, confirmation of the diagnosis was given by a neurologist. The neurological examinations were normal in all patients.

### Inclusion and exclusion criteria

Inclusion criteria comprising the diagnosis of episodic migraine without aura were confirmed by an experienced neurologist according to the International Classification of Headache Disorders, third edition (ICHD-IIIb) (Headache Classification Committee of the International Headache Society, 2013). Exclusion criteria were (a) current medical preventive therapy (patients were included if preventive medication was stopped at least 8 weeks prior to study participation), (b) other internal or neurological diseases, and (c) regular exercise experience within the last 6 months. The ability to participate in an intense exercise program was confirmed using the physical activity readiness questionnaire (PAR-Q) and the exercise test. Patients with CVD or acute or chronic inflammatory disease were not included in the study.

### Testing procedures

#### Arterial stiffness

Arterial stiffness parameters were obtained using an oscillometric Mobil-O-Graph® PWA Monitor device (I.E.M GmbH, Stolberg, Germany) with integrated ARCSolver® software. The blood pressure cuff was placed on the left upper arm while the patient was lying in a resting supine position. The measurements of arterial stiffness and central hemodynamics using the oscillometric method stand in good agreement with the conventional tonometric method (Wassertheurer et al., 2010). The first of three measurements was performed after 5 min of resting period. The two following measurements were performed at 2-min intervals. From the measurements, central blood pressures, crude Aix, and AIx@75 as well as PWV were extracted. After data readout, every measurement was reviewed for erroneous values. The mean and standard deviation of three valid measurements were calculated.

#### Recording migraine days

Prior to training (4-week run-in period) and during the exercise intervention a standardized paper and pencil migraine diary was kept by all patients (Baos et al., 2005). The last 4 weeks of the intervention period were considered as post value.

#### Maximal exercise testing

Exercise testing was conducted on a treadmill (HP Cosmos Pulsar, H/P/COSMOS Sports & Medical, Germany) to determine the individual anaerobic lactate-threshold (LT), maximal heart rate (HR_max_), and VO_2max_. To obtain these data within one test, incremental protocols were combined with a ramp-like protocol (Meyer et al., 2007). Patients started at 5 km/h, step duration lasted 3 min with an increment of 1 km/h and a capillary lactate withdrawal for each step. After 5 steps (9 km/h), a ramp-wise increase of 1.0 km/h per minute without lactate withdrawal was applied to assess VO_2max_ (Scharhag-Rosenberger et al., 2012). During exercise testing, breath-by-breath spirometric gas-exchange data (Metamax 3b, Cortex, Leipzig, Germany), heart rate (HR) (Polar Electro Oy, Kempele, Finland), and ratings of perceived exertion (RPE) (Borg, 1998) were collected. The maximum of the three highest consecutive oxygen uptake and heart rate values were regarded as VO_2max_ and HR_max_, while the individual anaerobic LT was determined according to Hagberg and Coyle (1983). For the analyses, the running speed at the LT in kilometer per hour (km/h) was used.

#### Exercise intervention

Both HIT and MCT were conducted individually under supervision of a sports scientists at the Department of Sports, Exercise and Health (DSBG) of the University of Basel, Switzerland, and consisted of an individual running program on a treadmill. A general warm-up of 400 m easy running followed by two skipping exercises and a cool-down period of 400 m and stretching exercises were included. MCT was performed by maintaining the calculated target heart rate of 70% (± 5 beats per minutes, bpm) of HR_max_ for 45 min (Helgerud et al., 2007). Exercising 45 min at this specific intensity results in similar energy expenditure as the chosen HIT program (Helgerud et al., 2007). During HIT, the target intensity of 90 to 95% HR_max_ (±5 bpm) was generally reached after 1 min. Each interval lasted 4 min, followed by an active rest period of 3 min at 70% of HR_max_. The 4-min intervals were repeated four times (Helgerud et al., 2007). HR-monitors collected heart rate data of each training session. The total training distance was recorded for each session. In total, 24 training sessions were conducted. It was required to complete at least 20 training sessions (~80% attendance, “per protocol” analysis) during the 12-week training period. No adverse and serious adverse events were reported.

#### Statistics

Indices of central haemodynamics and arterial stiffness, migraine attack frequency, and maximal and submaximal fitness were given as means with standard deviations (*SD*).

Analyses of covariance (ANCOVA) were computed to adjust between-group effects for potential baseline differences (Vickers and Altman, 2001). To estimate practical relevance of the ANCOVA between group effects, effect sizes (partial eta squared, $\eta_{p}^{2}$) were additionally calculated. According to Cohen (Cohen et al., 2013), an $\eta_{p}^{2}$ ≥ 0.01 indicates a small, ≥0.06 a medium and ≥0.14 a large effect. Standardized mean differences (Cohen's d, trivial: SMD < 0.2, small: 0.2 ≤ SMD < 0.5, moderate: 0.5 ≤ SMD < 0.8, large SMD ≥ 0.8; Cohen, 1992) were calculated for each pairwise comparison for each variable.

Additionally, the absolute and percentage differences as well as the standardized mean differences (Cohen's d) in the change scores between HIT, MCT, and CON from pre- to post-testing were also calculated together with 90% confidence intervals according to the magnitude-based inference approach (Batterham and Hopkins, 2006): These calculations were adjusted for pre-test values as well. A practically worthwhile change was assumed when the difference score was at least 0.2 of the between-subject standard deviation (Hopkins et al., 2009). The probability for an effect being practically worthwhile was calculated according to the magnitude-based inference approach using the following scale: 25–75%, possibly; 75–95%, likely; 95–99.5%, very likely; >99.5%, most likely (Batterham and Hopkins, 2006). The default probabilities for declaring an effect practically beneficial were <0.5% (most unlikely) for harm and >25% (possibly) for benefit (Hopkins et al., 2009). All calculations were conducted using a published spreadsheet in Microsoft® excel (Hopkins, 2007).

## Results

### Arterial stiffness

**Figure 2:**
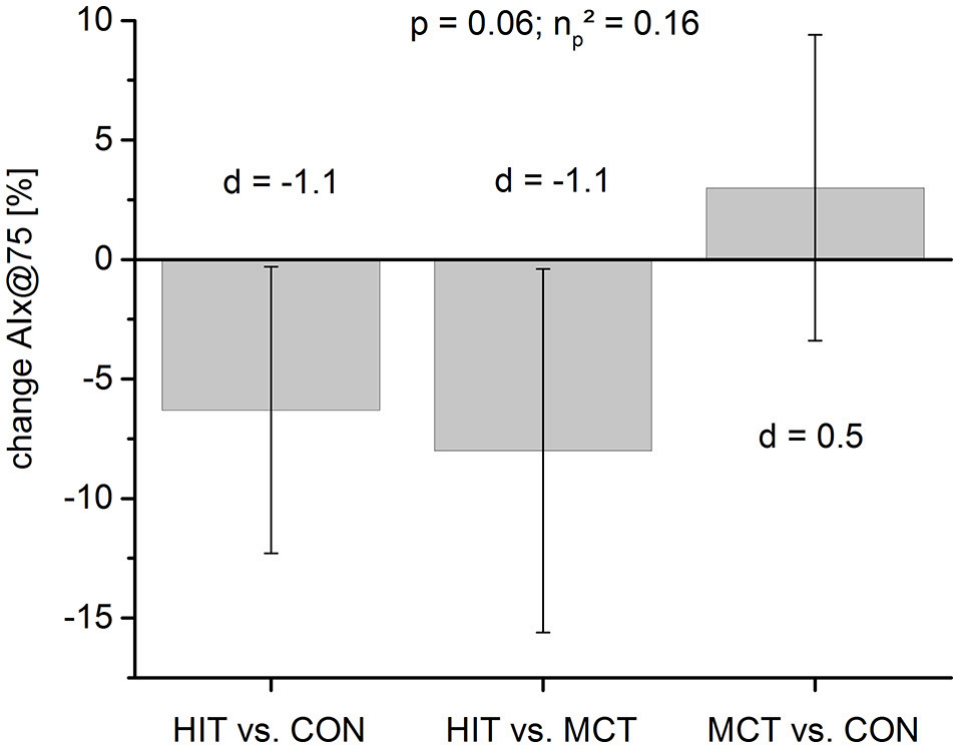
Mean change scores (%) for peripheral arterial stiffness (AIx@75) for pairwise between group comparison. Standardized mean differences are given as Cohen's d indicating moderate effects for HIT (0.5 < d < 0.7) compared to both groups. MCT vs. CON remains unchanged.

Taking baseline values into account, ANCOVA revealed a large and relevant between group effect for AIx@75 (*p* = 0.06, $\eta_{p}^{2}$ = 0.16). Pairwise comparison of AIx@75 showed moderate effects in HIT [pre: 22.0 (9.7), post: 14.9 (13.0), SMD = 0.62] with a likelihood of a meaningful effect of 85% likely beneficial compared to CON and 91% likely beneficial compared to MCT (Tables 2, 3). Figure 2 demonstrates the pairwise between group comparisons for AIx@75 change scores. Central systolic blood pressure (cSBP) was lowered in HIT [cSBP HIT: pre: 118.1 (23.4), post: 109.8 (15.7), SMD = 0.42] with a likelihood of a meaningful effect of 59% possibly beneficial compared to CON. Central diastolic blood pressure showed moderate effects in MCT [pre: 77.8 (6.8), post: 73.7 (6.6), SMD = 0.61] with a likelihood of a meaningful effect of 60% possibly beneficial compared to CON (Tables 2, 3).

**Table 2:** Pre and post intervention results of all three groups for peripheral and central vessel parameters and arterial stiffness parameters.

		**Pre mean (*SD*)**	**Post mean (*SD*)**	**SMD**	**ANCOVA**	
					***p***	**$\eta_{p}^{2}$**
Migraine days [Days/Month]	HIT	3.8 (3.0)	1.4 (1.2)	1.05	0.13	0.13
	MCT	4.2 (2.2)	3.1 (2.9)	0.43		
	CON	3.2 (2.4)	2.0 (1.6)	0.59		
pSBP [mmHg]	HIT	118.8 (8.1)	115.6 (9.3)	0.37	0.57	0.03
	MCT	117.7 (10.2)	118.0 (13.3)	−0.03		
	CON	121.8 (11.1)	120.7 (10.0)	0.10		
pDBP [mmHg]	HIT	78.7 (5.9)	76.8 (7.4)	0.28	0.31	0.07
	MCT	76.8 (6.7)	73.2 (6.4)	0.55		
	CON	78.3 (7.6)	77.4 (7.4)	0.12		
pPP [mmHg]	HIT	40.1 (4.6)	38.4 (4.8)	0.36	0.31	0.07
	MCT	40.9 (7.3)	40.3 (7.5)	0.08		
	CON	43.5 (6.2)	43.4 (5.1)	0.02		
cSBD [mmHg]	HIT	118.1 (23.4)	109.8 (15.7)	0.42	0.50	0.04
	MCT	109.8 (9.1)	107.0 (8.0)	0.33		
	CON	113.7 (10.8)	113.1 (11.3)	0.05		
cDBD [mmHg]	HIT	79.1 (5.9)	78.2 (11.4)	0.10	0.33	0.06
	MCT	77.8 (6.8)	73.7 (6.6)	0.61		
	CON	79.1 (7.7)	78.3 (7.5)	0.11		
cPP [mmHg]	HIT	32.7 (5.6)	31.7 (7.2)	0.16	0.42	0.05
	MCT	32.0 (5.0)	33.3 (4.4)	−0.28		
	CON	34.6 (6.1)	34.9 (6.2)	−0.05		
AIx [%]	HIT	28.2 (10.1)	23.8 (13.4)	0.37	0.13	0.12
	MCT	21.9 (8.7)	28.8 (12.7)	−0.63		
	CON	23.6 (16.9)	24.9 (13.4)	−0.09		
AIx@75 [%]	HIT	22.0 (9.7)	14.9 (13.0)	0.62	0.06	0.16
	MCT	16.6 (8.5)	21.3 (10.4)	−0.49		
	CON	17.9 (15.9)	18.8 (12.5)	−0.06		
PWV [m/s]	HIT	5.9 (1.0)	5.9 (1.2)	0	0.79	0.01
	MCT	5.8 (0.9)	5.7 (0.8)	0.11		
	CON	6.0 (1.1)	6.0 (1.2)	0		
VO_2max_ [ml/min/kgBW]	HIT	36.8 (5.2)	41.3 (8.3)	−0.65	0.13	0.12
	MCT	36.9 (5.1)	38.3 (6.1)	−0.25		
	CON	36.2 (6.3)	36.3 (5.7)	−0.02		
IAT [km/h]	HIT	8.2 (0.8)	8.7 (0.6)	−0.71	0.08	0.15
	MCT	8.3 (1.0)	8.4 (1.0)	0.10		
	CON	8.5 (1.1)	8.3 (1.2)	0.17		

*All results depicted as mean and standard deviation (SD). pSBP, peripheral systolic blood pressure; pDBP, peripheral diastolic blood pressure; pPP, peripheral pulse pressure; cSBP, central systolic blood pressure; cDBP, central diastolic blood pressure; cPP, central pulse pressure; AIx, Augmentationindex; AIx@75, Augmentationindex corrected for 75 beats per minute; PWV, pulse wave velocity*.

**Table 3:** Parallel Group Trials for HIT, MCT, and CON.

**Maximal parameters**	**Differences in means**	**Standardized mean difference [90% CI]**	**Probability for a practically worthwhile effect**
**pSBP [mmHg]**			
HIT vs. CON	−2.0 [−5.6; 1.6]	−0.19 [−0.54; 0.15]	51%; possibly beneficial
MCT vs. CON	3.5 [−4.1; 11.1]	0.30 [−0.36; 0.97]	11%; unlikely beneficial
HIT vs. MCT	−3.3 [−10.5; 4.0]	−0.34 [−1.09; 0.42]	64%; possibly beneficial
**pDBP [mmHg]**			
HIT vs. CON	−1.1 [−5.0; 2.7]	−0.16 [−0.71; 0.38]	47%; possibly beneficial
MCT vs. CON	−2.0 [−5.9; 1.8]	−0.27 [−0.77; 0.24]	61%; possibly beneficial
HIT vs. MCT	1.8 [−0.9; 4.6]	0.27 [−0.14; 0.69]	3%; very unlikely beneficial
**pPP [mmHg]**			
HIT vs. CON	−1.5 [−4.4; 1.4]	−0.25 [−0.73; 0.24]	58%; possibly beneficial
MCT vs. CON	−0.3 [−3.8; 3.3]	−0.04 [−0.53; 0.45]	30%; possibly beneficial
HIT vs. MCT	−1.3 [−4.6; 2.0]	−0.20 [−0.72; 0.31]	52%; possibly beneficial
**cSBP [mmHg]**			
HIT vs. CON	−4.7 [−13.0; 3.5]	−0.24 [−0.67; 0.18]	59%; possibly
MCT vs. CON	−2.9 [−6.5; 0.8]	−0.27 [−0.61; 0.08]	66%; possibly
HIT vs. MCT	0.0 [−8.2; 8.2]	0.00 [−0.42; 0.42]	22%; unlikely
**cDBP [mmHg]**			
HIT vs. CON	−2.3 [−7.9; 3.4]	−0.32 [−1.11; 0.47]	61%; possibly beneficial
MCT vs. CON	−2.0 [−6.2; 2.1]	−0.26 [−0.80; 0.28]	60%; possibly beneficial
HIT vs. MCT	3.8 [0.1; 7.6]	0.57 [0.01; 1.14]	2%; very unlikely
**cPP [mmHg]**			
HIT vs. CON	−1.3 [−4.8; 2.2]	−0.21 [−0.78; 0.36]	53%; possibly beneficial
MCT vs. CON	1.9 [−1.2; 5.1]	0.33 [−0.20; 0.85]	5%; unlikely beneficial
HIT vs. MCT	−2.1 [−4.8; 0.7]	−0.37 [−0.87; 0.13]	74%; possibly beneficial
**AIx [%]**			
HIT vs. CON	−4.4 [−10.6; 1.9]	−0.30 [−0.72; 0.13]	76%; possibly beneficial
MCT vs. CON	4.5 [−3.1; 12.2]	0.32 [−0.22; 0.86]	6%; unlikely beneficial
HIT vs. MCT	−7.8 [−16.6; 0.9]	−0.74 [−1.58; 0.09]	87%; likely beneficial
**AIx@75 [%]**			
HIT vs. CON	−6.3 [−12.2; −0.3]	−0.45 [−0.88; −0.02]	85%; likely beneficial
MCT vs. CON	3.0 [−3.4; 9.4]	0.23 [−0.25; 0.70]	7%; unlikely beneficial
HIT vs. MCT	−8.0 [−15.6; −0.3]	−0.80 [−1.56; −0.03]	91%; likely beneficial
**PWV [m/s]**			
HIT vs. CON	0.0 [−0.2; 0.2]	0.04 [−0.15; 0.23]	2%; very unlikely beneficial
MCT vs. CON	0.0 [−0.2; 0.1]	−0.05 [−0.16; 0.07]	3%; very unlikely beneficial
HIT vs. MCT	0.1 [−0.1; 0.3]	0.07 [−0.12; 0.27]	2%; very unlikely beneficial
**VO**_2max_ **[ml/min/kgBW]**			
HIT vs. CON	4.7 [0.2; 9.3]	0.79 [0.04; 1.54]	91%; likely beneficial
MCT vs. CON	1.5 [−1.1; 4.1]	0.25 [−0.19; 0.69]	60%; possibly beneficial
HIT vs. MCT	3.1 [−1.2; 7.4]	0.57 [−0.22; 1.37]	80%; likely beneficial
**IAT [km/h]**			
HIT vs. CON	1.1 [0.5; 1.6]	1.07 [0.52; 1.61]	99%; very likely beneficial
MCT vs. CON	0.7 [0.0; 1.5]	0.68 [0.00; 1.36]	89%; likely beneficial
HIT vs. MCT	0.3 [−0.1; 0.8]	0.39 [−0.12; 0.89]	75%; likely beneficial
**Migraine Days [Days/Month]**			
HIT vs. CON	−0.8 [−1.7; 0.0]	−0.28 [−0.58; 0.02]	71%; possibly beneficial
MCT vs. CON	0.3 [−0.9; 1.5]	0.13 [−0.34; 0.60]	42%; possibly beneficial
HIT vs. MCT	−1.5 [−2.6; −0.3]	−0.54 [−0.95; −0.12]	92%; likely beneficial

*Difference in means as well as standardized mean differences (90% confidence intervals) are given for peripheral and central haemodynamic parameters as well as for arterial stiffness. The probability for an effect being practically beneficial was calculated according to the magnitude-based inference method*.

PWV showed no changes in any of the three groups (Tables 2, 3).

### Migraine days

ANCOVA, again taking baseline values into account, revealed moderate and relevant between group effect for migraine days (*p* = 0.13, $\eta_{p}^{2}$ = 0.13). Pairwise comparison of migraine days showed very large effects in HIT [pre: 3.8 (3.0), post: 1.4 (1.2), SMD = 1.05] with a likelihood of a meaningful effect of 71% possibly beneficial compared to CON and 92% likely beneficial compared to MCT. MCT revealed a 42% possibly beneficial effect compared to CON. Migraine days are reported as number of migraine days per month (Tables 2, 3).

### Maximal and submaximal fitness parameters

ANCOVA revealed a moderate but relevant between group effect for VO_2max_ (*p* = 0.13, $\eta_{p}^{2}$ = 0.12) and IAT (*p* = 0.08, $\eta_{p}^{2}$ = 0.15). Pairwise comparison of VO_2max_ showed moderate effects in favor of HIT [(ml/min/kg) pre: 36.8 (5.2), post: 41.3 (8.3), SMD = −0.65] and small effects for MCT [(ml/min/kg) pre: 36.9 (5.1), post: 38.3 (6.1), SMD = −0.25]. HIT showed a 91% likely beneficial effect while MCT revealed a 60% possibly beneficial effect over CON. Pairwise comparison of IAT revealed moderate effects in HIT [(km/h) pre: 8.2 (0.8), post: 8.7 (0.6), SMD = −0.71].

## Discussion

The aim of the present randomized controlled trial was to investigate whether different aerobic exercise intensity modalities yield differential effects on central systolic and diastolic blood pressure, pulse wave reflection, and PWV in episodic migraineurs. To the best of our knowledge, no study to date has compared the effects of different exercise modalities and intensities on arterial stiffness and migraine. Our results show that HIT but not MCT was able to lower AIx@75 while both exercise programs were able to reduce migraine days and improve physical fitness. We could further elucidate that HIT is superior to MCT in improving AIx as an indirect marker of systemic arterial stiffness, which indicates cardiovascular risk reduction in migraineurs. The exercise-induced adaptations of arterial stiffness may be attributed in large part to an increased availability of nitric oxide (NO) and a consecutive improvement of endothelial function. Increases in shear stress during exercise induce NO production of the endothelium, which results in relaxation of vascular smooth muscle cells, arterial vasodilation, and a drop in vascular resistance (Alsop and Hauton, 2016). It has been suggested that the regulation of vascular tone in response to endothelial sheer stress stimulus is impaired in migraineurs and NO is regarded as crucial factor in the process (Buse et al., 2017). An increased nitrate-mediated response during development of migraine has been suggested, supporting the theory of overregulated NO sensitivity in migraineurs (Buse et al., 2017). Our results indirectly suggest that the improvement of NO regulation is intensity-dependent, whereby higher intensities applied in intervals compel stronger vascular adaptations. These findings correspond to previous findings comparing acute effects of HIT and MCT on arterial stiffness, which showed that AIx@75 declined significantly over time after HIT but less after MCT (Hanssen et al., 2015). HIT induces higher laminar shear stress compared to lower continuous exercise intensities and, therefore, HIT represents a stronger stimulus for shear stress-induced release of NO (Adams et al., 2017). This may, in part, explain why HIT reduced AIx@75 as well as migraine days more efficiently than MCT. Regular exercise, and HIT in particular, has the potential to improve NO production and regulation in migraineurs and reduce migraine days. Further research is needed to clarify the postulated mechanistic link and its causality.

Interestingly, PWV showed no changes in any of the three groups even though HIT reduced central systolic blood pressure while MCT reduced central diastolic blood pressure. These results are in line with the few studies which have examined the effects of exercise on PWV (Heffernan et al., 2007; Munir et al., 2008). Central arteries seem to require longer intervention periods with regular stimulus than the conduit and more peripheral vessels in order to adapt to aerobic exercise stimuli (Hanssen et al., 2015). As expected, CON underwent no changes in any arterial stiffness parameters.

Migraine attacks are accompanied by repeated spells of vascular inflammation and repeated attacks of migraine have been suggested to induce inflammatory changes of cranial arteries (Bolay et al., 2002). These recurring inflammatory processes may predispose to vascular endothelial dysfunction (Schillaci et al., 2010). In previous studies, HIT has been shown to be superior to MCT in reducing arterial stiffness over a 24 h period in normotensive patients (Hanssen et al., 2015) as well as reducing cardio-metabolic risk in patients with CVD (Wisløff et al., 2007). The improvement of AIx@75 following HIT in our study indicates the potential to reduce cardiovascular risk in migraineurs, as a decrease in AIx is associated with a decreased cardiac afterload and left ventricular burden long-term (Vlachopoulos et al., 2012; Hanssen et al., 2015). A 10% increase of AIx is associated with a 30% increased relative risk for cardiovascular events (Vlachopoulos et al., 2010b). Therefore, HIT may prove to be an effective therapy for the reduction of migraine symptoms, improving vascular function and integrity as well as reducing cardiovascular risk. We would like to postulate that interval-like application of high intensities evoke patterns of wave reflection that improve vascular function more than continuous lower intensity applications. Increased NO bioavailability evoked by the HIT protocol is one of the most likely mechanisms that help explain these findings.

The study comprises some limitations that need to be mentioned. The sample size of the pilot study might be considered low. In our group analysis, we refrained from interpreting our data on the basis of mere conventional *p*-values to estimate relevant between-group effects, as *p*-values do not sufficiently allow for continuous estimation of relevant interventional effect sizes (Pocock and Stone, 2016). Indeed, these would have failed to reach significance due to the relatively small sample size of this study. Although increased arterial stiffness and cardiovascular risk has been reported in migraine, the patients in our study did not have impaired baseline levels of AIx or PWV according to the current recommendations for normal values (Hansen, 2010; Janner et al., 2010). A selection bias may have occurred whereby healthier and physically fitter patients were motivated to participate in the exercise intervention trial. In addition, despite group allocation based on physical fitness and migraine days per month, notable baseline differences in migraine days as well as arterial stiffness parameters AIx and AIX@75 need to be addressed. These findings might further be caused by the dropouts. We applied fitness and the MIDAS (migraine disability assessment) questionnaire as strata for group allocation. Moreover, we included baseline values and age as covariates. Thus, our results are adjusted for potential baseline differences.

## Conclusion

Regular aerobic exercise has beneficial effects on blood pressure, vascular function, and cardiovascular risk. Since HIT was more effective in reducing systemic arterial stiffness and migraine days than MCT, we postulate that the exercise effects in migraine patients depend on intensity and modality of the exercise therapy. HIT should be considered as a complementary treatment strategy for migraine treatment and prevention. It is all the more important since drug treatment of migraine is oftentimes addressed with precautions in the presence of cardiovascular comorbidities. Exercise therapy seems to have the potential to play a crucial role in future preventive strategies to combat migraine complaints and development of associated CVD.

## Author contributions

Study design: HH, TS, and LD; data collection: AM, SM, AR, AP, and CK; statistical analysis: AM and LD; manuscript preparation: HH, AM, TS, and LD; manuscript revision: OF, AS-T, and LZ.

### Conflict of interest statement

The authors declare that the research was conducted in the absence of any commercial or financial relationships that could be construed as a potential conflict of interest.
